# Gold nanoparticle assembly on porous silicon by pulsed laser induced dewetting

**DOI:** 10.1039/d0na00043d

**Published:** 2020-01-31

**Authors:** Alison Joy Fulton, Vinayaraj Ozhukil Kollath, Kunal Karan, Yujun Shi

**Affiliations:** Department of Chemistry, University of Calgary Calgary AB T2N 1N4 Canada shiy@ucalgary.ca +1-403-2108674; Department of Chemical and Petroleum Engineering, University of Calgary Calgary AB T2N 1N4 Canada

## Abstract

This work reports the influence of the substrate in the pulsed laser-induced dewetting (PLiD) of Au thin films for the fabrication of nanoparticle (NP) arrays. Two substrates were studied, *i.e.*, polished silicon and porous silicon (PS), the latter being fabricated *via* electrochemical anodization in HF-containing electrolytes. The effect of both PLiD and substrate preparation parameters was explored systematically. On polished silicon substrates, it has been shown that uniform, randomly arranged NPs between 15 ± 7 nm and 89 ± 19 nm in diameter are produced, depending on initial thin film thickness. On PS however, there are topographical features that lead to the formation of ordered NPs with their diameters being controllable through laser irradiation time. The presence of surface pores and the appearance of surface ripples under low HF concentrations (<9.4 wt%) during electrochemical anodization results in this unique dewetting behaviour. Through AFM analysis, it has been determined that the ordered NPs sit within the valleys of the ripples, and form due to the atomic mobility enabled using the PLiD approach. This work has demonstrated that the utilization of topographically complex PS substrates results in size controllable and ordered NPs, while the use of polished Si does not enable such control over array fabrication.

## Abstract 2

This work reports the influence of the substrate in the pulsed laser-induced dewetting (PLiD) of Au thin films for the fabrication of nanoparticle (NP) arrays.

## Graphical abstract

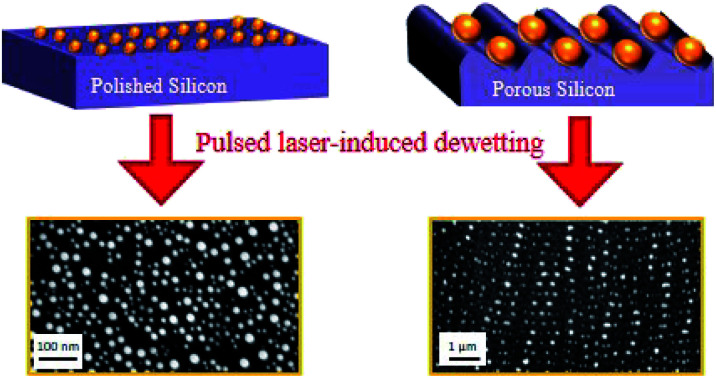

## 1. Introduction

Porous silicon (PS) has been widely used as a versatile material in many applications due to its large surface areas as well as excellent optical, electrical and mechanical properties.^1^ PS can be readily synthesized using electrochemical etching in the presence of HF, providing tunable morphology depending on the etching conditions and the intrinsic properties of silicon.^2^ The use of PS as a host material for metallic nanostructure and nanoparticle (NP) deposition has recently gained extensive research interest since the composite structures bring the properties of both metal nanoparticles and PS into play. These metal NP–PS systems have therefore, been studied for improving their sensing^3,4^ and luminescent^5–7^ properties. PS coated with noble metal NPs, such as Au and Ag, has also been extensively studied for surface enhanced Raman scattering (SERS) substrates.^8–10^

The most commonly used methods for the deposition of metal NPs on PS are the wet chemical techniques, including drop casting of metal colloidal solutions,^3,5,6^ metal-assisted chemical etching of silicon,^4,11^ immersion plating,^9,10,12^ electroplating,^7,13^ and thermal decomposition of metal salts.^14^ These wet chemical methods are often simple, inexpensive, and applicable for high throughput production, however they possess certain drawbacks including long synthesis time, inhomogeneous NP loading, and NP aggregation. For example, the use of colloidal solutions generally results in high concentrations of metal NPs in the ring around the dried drop with a lack of homogeneity across the sample as a whole.^6,8^ The use of a stabilizing agent to prevent NP agglomeration through charge repulsion can limit the applicability of the metal NP–PS system for sensory applications to analyte molecules of the same-sign charge.^15^ The electrochemical methods typically result in metal deposition primarily at the pore tips or walls rather than on the surface.^16–18^ While this is advantageous towards increasing the electrical conductivity of the PS for use in integrated circuits, it is disadvantageous for applications in which surface limited Au NPs are desired.

Methods other than the wet chemical ones are not well explored in the literature for forming metal NPs on PS substrates. Reports have been found using physical vapor deposition (PVD) forming Au nano-grains on PS.^19,20^ Recently, Lu *et al.*^21^ and Wang *et al.*^22^ reported the novel use of solid-state dewetting of Au films induced by thermal annealing to produce neat Au nanoaperture arrays on templated Si substrates. Thermal dewetting has also been used to obtain ordered arrays of Au NPs on topographically modified substrates patterned with arrays of inverted pyramid-shape pits in Si wafers.^23^ The thermal dewetting process is robust and simple. However, substrate deformation due to the extended exposure at high temperatures needed for the dewetting of high-melting-point metals limits its use mainly to low-melting-point metals.^24,25^ In addition, there are potential metal–substrate chemical interactions and metal diffusion into the substrate, complicating the dewetting process.^26^ An alternative dewetting technique is liquid-state dewetting induced by pulsed laser irradiation. Pulsed laser induced dewetting (PLiD) of thin metallic films causes a break-up of films into NPs at a laser fluence above the melting threshold of the metal.^26,27^ The optical energy is absorbed by the electrons of the metal and these excited electrons in the conduction band collide with lattice phonons and other electrons resulting in the release of energy, in the form of heat. This process, leading to a temperature increase within the metal and subsequent melting, occurs in approximately 0.1–1 ps.^28,29^ Metal re-solidification occurs within approximately 10 ps.^28^ This time scale ensures that rapid melting and re-solidification processes occur within a single nanosecond laser pulse,^26^ with the width typically in the range of 6–12 ns. Furthermore, PLiD of metallic thin films on substrates containing an oxide layer, such as Si with its native SiO_2_, typically leads to the thermal energy being confined almost completely within the metal films. This is because SiO_2_ has low thermal conductivity, meaning that only a small amount of the laser-generated thermal energy is dispersed through to the Si substrate beneath it.^29^ As a result, the PLiD method prevents any detrimental effect to the underlying substrates, thanks to the instantaneous heating within the short width of laser pulses. It is therefore suitable for making NPs of high-melting-point metals such as Pt,^30^ Fe,^31^ and Co–Pt,^32^ in addition to low-melting-point metal NPs, *e.g.*, Au,^24,33^ Ag,^33,34^ and Cu.^27,35^

PLiD *via* liquid-state dewetting is able to produce spatially ordered metal NPs, which have more uniform distributions than those obtained from solid-state thermal dewetting.^32^ Furthermore, NPs formed *via* PLiD conform to the topography of the substrate, allowing extra controls on the spatial order and shape of these NPs if patterned substrates are used.^24,32,36^ Various attempts have been made to generate ordered metal NP arrays *via* dewetting processes using patterned substrates. Lithography is an excellent method to provide precise control in the templated substrate patterns.^22,23,32^ However, this method requires expensive facilities and the implementation is time consuming. Electrochemical anodization of metals is known to produce self-ordered arrays of porous material. By using PLiD of metallic thin films on these nanopatterned substrates, metal NP arrays have been successfully produced using dimpled Ta templates formed by anodization.^24,30^ In addition, highly ordered TiO_2_ nanotube arrays *via* anodization have been used as a substrate for thermal dewetting of Au ^37^ and Cu ^38^ thin films. In general, substrates prepared by electrochemical anodization patterning methods are advantageous for use in dewetting of thin metallic films because the anodization results in an oxide layer on the surface, which is important for the thermodynamic instability leading to spontaneous dewetting of films to form NPs.^39,40^

Although PS formation by electrochemical anodization has been extensively studied, providing a high-level control over the pore size and shape, the use of PS as a substrate in PLiD of metallic films has remained relatively unexplored. In this work, we report an effective method to produce Au NP assemblies on electrochemically anodized PS substrates *via* PLiD. It is shown that seemingly disordered porous n-type silicon substrates do in fact possess certain degrees of order, though they may not be initially apparent using typical top-down imaging analysis methods. The resulting NP arrays are very stable and do not exhibit any reorganization or agglomeration over time. For comparison, a study of the PLiD of Au on polished silicon substrates is also presented. The use of PLiD to fabricate Au NPs on the surface of PS provides a rapid alternative to wet chemical method without the loss of material within the pores and a high level of homogeneity across the sample as a whole.

## 2. Results and discussion

### 2.1 Pulsed laser induced dewetting of thin Au films on polished Si wafers

The behavior of PLiD of thin Au films was first studied on polished Si wafers, which has a native SiO_2_ layer of 3 nm, as confirmed by ellipsometry. The thickness of this native oxide layer was found to remain relatively constant following laser irradiation of a plain Si wafer with no Au thin film, indicating that laser irradiation does not affect the substrate morphology. This was further confirmed through FESEM. The dependence of the produced Au NP morphology on various parameters, including laser fluence, laser irradiation time, and thin film thickness, has been systematically explored. In this work, PLiD was carried out under ambient conditions as Au does not readily form an oxide.^25^ This is confirmed by our energy-dispersive X-ray (EDX) spectroscopy analysis of the two samples of Au thin films dewetted in vacuum and in ambient air, which showed no difference in the oxygen signals.

**Fig. 1:**
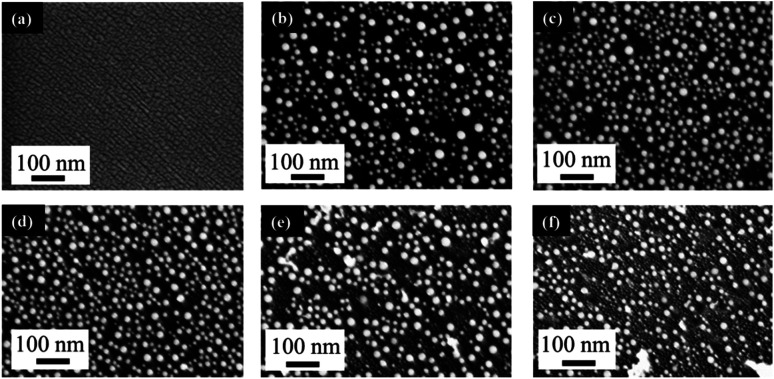
FESEM images comparing the effect of laser fluence in the PLiD of 2.9 nm Au thin films on polished Si wafer. All samples were dewetted with one laser pulse with varying fluences at (a) 26 mJ cm^−2^, (b) 101 mJ cm^−2^, (c) 200 mJ cm^−2^, (d) 325 mJ cm^−2^, (e) 520 mJ cm^−2^, and (f) 702 mJ cm^−2^. The scale bars are the same for all images.

We first examined the effect of laser fluence with the aim of determining the threshold fluence. For this, Au films of a thickness of 2.9 nm were used. Laser fluence was varied from 26 mJ cm^−2^ to 702 mJ cm^−2^. Fig. 1 shows the FESEM images of Au NPs generated on polished Si with one laser pulse at varying fluences. At the two lowest fluences of 26 and 49 mJ cm^−2^, no dewetting occurs. The morphology in Fig. 1a after irradiation with a laser fluence of 26 mJ cm^−2^ is nearly identical to the un-irradiated, as-deposited Au thin film on SiO_2_/Si, which showed discontinuous formation of islands. The deposition of Au thin films on insulators is known to follow the Volmer–Weber model,^41,42^ where 3-dimensional island growth dominates. This model predicts that thinner films will have a higher level of discontinuity, which is seen in the 2.9 nm thick Au film in Fig. 1a. At the next higher fluence of 75 mJ cm^−2^, full dewetting was observed with an average NP diameter of 17.0 ± 3.8 nm, indicating a threshold fluence between 49 and 75 mJ cm^−2^. Continuing increase in the laser fluence from 101 mJ cm^−2^ (Fig. 1b) to 200 mJ cm^−2^ (Fig. 1c), then further to 325 mJ cm^−2^ (Fig. 1d) resulted in a slight decrease of NP size from 16.3 ± 4.0 nm to 15.8 ± 3.1 nm, then to 13.0 ± 2.5 nm, respectively. At fluences of 520 and 702 mJ cm^−2^, which are much greater than the threshold fluence, the size distributions of the obtained Au NPs become less uniform, with an increasing number of small NPs being seen in addition to the large NPs that dominate in the sample (Fig. 1e and f). This is in contrast to the monodispersed behavior seen at relatively low laser fluences (Fig. 1b–d). These small NPs were difficult to measure accurately and the size distributions of 14.9 ± 3.0 nm for Fig. 1e and 14.0 ± 3.2 nm for Fig. 1f were obtained from the large NPs only. The average NP diameters would be lower if the smaller NPs could be included in the distributions. The decreasing trend in metal NP sizes with increasing fluence has been observed in PLiD of other systems, including Pt on Ta_2_O_5_/Ta ^30^ and Ag on SiO_2_.^40^ According to Henley *et al.*, this size decrease can be attributed to the fragmentation of larger NPs into smaller NPs at higher temperatures.^40^ Interestingly, similar observations of increasing number of smaller NPs at higher fluences were obtained in their work on dewetting both Au and Ag thin films on SiO_2_ substrates.

Next, the effect of laser irradiation time, or number of laser pulses, on the morphology of the NPs was explored. For this, a thin film of 2.9 nm Au was dewetted at 200 mJ cm^−2^ with irradiation times ranging from 1 to 200 pulses. The morphology of Au NPs at different irradiation times is similar to the one presented in Fig. 1c at one laser pulse. Over this irradiation time range, all metal NPs show unimodal distributions. From 1 pulse to 75 pulses, the NP diameter remains nearly constant, changing from 15.8 ± 3.1 nm (1 pulse) to 16.1 ± 3.6 nm (at 75 pulses). It is only at increased laser irradiation times (≥150 pulses) that a slight increase in NP diameter can be observed, with an increase from 18.5 ± 3.9 nm at 150 pulses to 20.5 ± 4.3 nm for 200 pulses. This trend is consistent with the one observed with the PLiD of Pt on Ta_2_O_5_/Ta, which showed a strong correlation between laser irradiation time and NP diameter.^30^ In both cases, the NP diameters increase with increasing laser irradiation time, however the increase is less pronounced in this work as the time span is much shorter here. This increase is due to the surface mobility of the NPs facilitated at longer irradiation time, resulting in the agglomeration of the smaller NPs into larger ones. In addition, Ostwald ripening may be contributing to the formation of the larger NPs at the expense of the smaller ones in the dewetting process.^43^

**Fig. 2:**
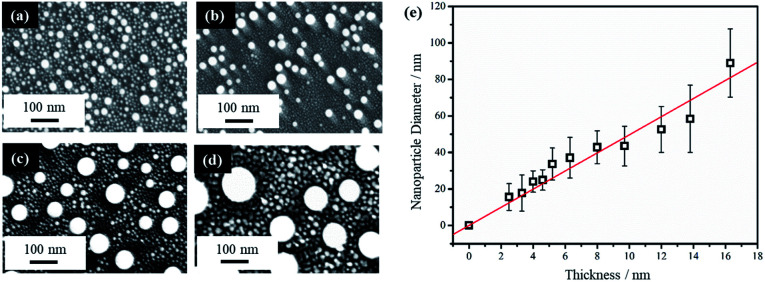
FESEM images of samples after PLiD of Au thin films on Si wafer of different thicknesses of (a) 2.5 nm, (b) 4.6 nm, (c) 12.0 nm, (d) 16.3 nm when dewetted with one laser pulse at 200 mJ cm^−2^, and (e) a plot of the average NP diameter as a function of initial thin film thickness. The scale bars are the same for all images.

Thin film thickness is a key factor in determining the NP morphology and whether a characteristic length scale has been developed during the liquid-state laser dewetting. We therefore, examined NP morphology dependence on film thickness. For this, the Au film thickness was varied from 2.5 nm to 16.3 nm. Fig. 2a–d show representative FESEM images for four samples of different thickness of 2.5, 4.6, 12.0, and 16.3 nm. All samples were irradiated by one laser pulse at a laser fluence of 200 mJ cm^−2^. It is apparent that there are nano-grains between the spherically shaped NPs that are more pronounced with the thicker Au films as seen in Fig. 2b–d. EDX analysis has confirmed that these nano-grains are Au. It has been confirmed that with increased irradiation time (10–100 pulses), these Au nano-grains are no longer present and well defined NPs alone are produced. EDX was also conducted on singular NPs and showed that they were composed of Au.

The average diameter of the large spherical NPs, excluding the small nano-grains, showed a linear increase with increasing thin film thickness. This is illustrated in Fig. 2e. Liquid-state dewetting is known to occur *via* nucleation or spinodal dewetting instability.^27,32,39^ In spinodal dewetting, a thin-film hydrodynamic instability leads to amplification of film thickness fluctuations and eventually ruptures the film, resulting in formation of NPs characterized by a well-defined length scale, which shows a quadratic dependence of inter-particle spacing (*L*) on the film thickness (*h*), *i.e.*, *L* ∝ *h*^2^. As a result, the NP diameter (*D*) shows a power dependence on *h*, following *D* ∝ *h*^5/3^. This relationship is not observed here, indicating a non-spinodal dewetting behavior. Nucleation and growth of holes can be initiated either homogenously at random locations or heterogeneously at surface defects. In this work, as shown in Fig. 1a, the Volmer–Weber growth model leads to nano-island formation in the as-deposited Au films, which could act as the nucleation sites, ultimately giving a well-defined length scale, represented by a linear dependence of NP diameter on the film thickness. Kojima *et al.* studied the electron-beam-induced dewetting of Au thin films on SiO_2_/Si substrates, where they found a similar linear relationship between the NP diameter and the Au film thickness in the range of 5 to 30 nm.^44^ No definitive reason was provided, although a surface tension gradient induced by a sharp thermal gradient in the surface layer was proposed as a possible contributor. On the other hand, Yadavali *et al.* observed a spinodal dewetting behavior in PLiD of Au films with a thickness from 3 to 16 nm deposited on a 400 nm-thick thermally grown SiO_2_ layer on top of polished Si wafers by electron beam deposition.^45^ This further supports that the deposition method and initial morphology of the thin films before dewetting are important in determining the dewetting pathways.

The NPs generated on polished Si wafer *via* PLiD range in diameter from 15 ± 7 nm by dewetting the lowest film thickness of 2.5 nm to 89 ± 19 nm from the thickest film of 16.3 nm studied in this work. In comparison to previous work by Yadavali *et al.* which reported an Au NP size of 116 ± 30 nm for a 12 nm thick Au film on SiO_2_/Si,^45^ the NPs produced here are smaller for the same-thickness films. Furthermore, compared to the broad size distributions of 10–60 nm obtained from dewetting a 2.8 nm Au thin films on chemically polished Ta substrates,^24^ more uniform and monodispersed Au NPs were obtained here. The reduction in NP size can be attributed to the initial surface roughness of the thin films prior to dewetting. Previous work on PLiD of Co metals on SiO_2_ substrates has clearly shown that the dewetting length scale, including NP size and inter-particle spacing, is significantly reduced with increasing film roughness.^39,46^ The surface roughness of the sputter-coated Au thin film in this work, characterized by atomic force microscopy (AFM), ranged from a root-mean-square roughness (*R*_rms_) value of 2.3 nm for a 2.9 nm thick film to a *R*_rms_ value of 1.1 nm for a thickness of 16.3 nm, which agreed with the prediction that thinner films gives rougher surface according to the Volmer–Weber growth model. The thin film growth model can be described in further detail according to the work by Ruffino *et al.* which describes the stages of Au film growth on Si substrates.^47,48^ Specifically, film growth begins with the nucleation of hemispherical Au clusters followed by lateral growth due to the adsorption of diffusing adatoms. As film deposition proceeds, lateral growth continues until full coverage is achieved. This is followed by vertical growth with the overall process being a conservative growth influenced by Au grain boundary diffusion. In Yadavali's work on PLiD of Au films on SiO_2_/Si, the surface roughness of their deposited films had an *R*_rms_ value lower than 0.5 nm for the entire thickness range of 3 to 16 nm.^45^ Therefore, the increased roughness in the initial films prior to dewetting is responsible for the much smaller NPs observed in this work. The importance of the results shown here is that it provides further support for the conclusion from PLiD of Co films on SiO_2_/Si that the surface roughness of the metal films can be used as an additional parameter, along with the conventionally used film thickness, to control the NP size.

### 2.2 Pulsed laser induced dewetting of thin Au films on porous silicon substrates

**Fig. 3:**
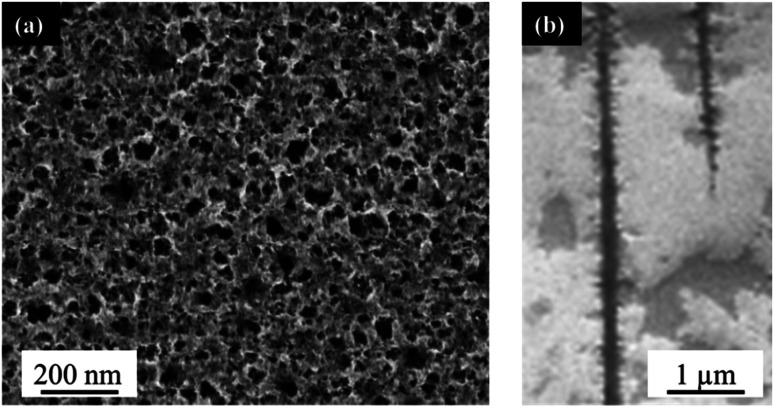
(a) Top-down, and (b) cross-sectional FESEM images of electrochemically anodized porous silicon in a 6.3 wt% HF solution at 15.1 V for 20 min.

The PLiD of thin Au films on the Si wafer substrates showed that the roughness of thin metallic film played an important role in determining the NP size on smooth substrates because of the imposed length scale resulting from the thin film deposition method and the observed Volmer–Weber growth model. In contrast to the formation of Au NPs on polished Si substrates, the use of porous Si (PS) substrates leads to significantly different results. This is because the presence of additional topographical features on templated or patterned substrates can further influence the formation and morphology of the NPs. Here, PS was fabricated using electrochemical anodization in HF-based electrolytes under high applied potentials as explained in detail elsewhere.^49^ Electrochemical anodization enables control over a variety of parameters, including HF concentration, applied voltage, and time, which can all be used to tune the pore sizes of the substrate. In this work, low-doped n-type Si was employed, which was the same as that used in the above mentioned studies on polished substrates (Section 2.1). The typical morphology of the produced PS can be seen in Fig. 3. Following anodization, a thin mesoporous (2–50 nm pore size) transition layer is formed on top of a macroporous (>50 nm pore size) layer. The pore size is influenced by the applied voltage and HF concentration. An increase in applied voltage leads to an increase in pore diameter, whereas an increase in HF concentration leads to a decrease in pore diameter. The mesoporous transition layer ranges in pore size from 35 ± 5 nm to 4 ± 1 nm with an increase in HF concentration from 3.2 wt% to 24.0 wt% HF at a constant voltage of 15.1 V for 20 min, with a representative sample prepared using a 6.3 wt% HF shown in Fig. 3a. The pores of the bulk macroporous region are significantly larger in diameter, ranging from 89 ± 9 nm at 9.1 V to 285 ± 28 nm at 16.1 V with a constant HF concentration (6.3 wt%) for 20 min, with Fig. 3b showing the one fabricated at 15.1 V. The inter-pore spacing for the macropores is between 1.2–2.1 μm, depending on the other anodization conditions.

In addition to pore size, HF concentration also plays a key role on the surface topography. Low HF concentrations (<9.4 wt% HF) produce a higher degree of surface roughening, in the form of surface ripples, with an *R*_rms_ value of 5.5 nm when using the 3.2 wt% HF. Higher HF concentrations (≥9.4 wt% HF) produce a smoother surface, with those prepared using 12.4 wt% HF giving an *R*_rms_ value of 3.6 nm. While these are merely comparative roughness values since they were obtained after fitting a parabolic flatten function in WSxM software, a polished silicon wafer has an *R*_rms_ value of 0.15 nm. This shows that while all HF concentrations enhance the roughness of the substrate, the effect is amplified at lower HF concentrations. This is related to the pore size, which increases with decreasing HF concentration. It is also related to the mechanism of dissolution as described in detail elsewhere.^49^ Specifically, at high HF concentrations, a divalent, direct dissolution mechanism is observed, whereas at low HF concentrations, a tetravalent, indirect dissolution mechanism is observed. This indicates that the dominant dissolution process, which is correlated to the HF concentration, impacts the surface roughness of the PS.

**Fig. 4:**
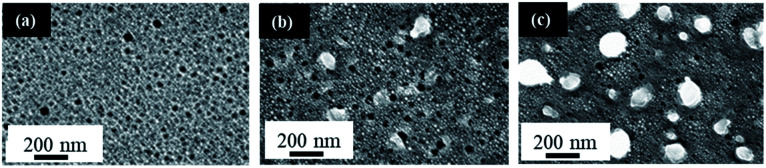
FESEM images of 3.0 nm Au thin films on PS (all prepared using 3.2 wt% HF at 15.1 V for 20 min) dewetted by using a 355 nm laser radiation at 200 mJ cm^−2^ with varying number of pulses: (a) 1 pulse, (b) 75 pulses, and (c) 150 pulses. All scale bars are the same.

Fig. 4 shows the SEM images of three samples of 3.0 nm Au thin film after being exposed to a 355 nm laser radiation at a fluence of 200 mJ cm^−2^ for various number of pulses in the range of 1–150 pulses on a PS substrate prepared using 3.2 wt% HF at 15.1 V for 20 min. It can be seen from Fig. 4a that a single laser pulse is sufficient to dewet the 3.0 nm Au thin film on PS. The resulting Au NPs are 9.0 ± 2.0 nm in diameter, which is even smaller than those (15.8 ± 3.1 nm) obtained when Au thin films of similar thickness (2.9 nm) on polished Si wafer were dewetted under the same conditions. This further supports that a rougher surface leads to smaller NPs. Fig. 4b indicates the onset of large NP formation (191 ± 32 nm diameter) at 75 pulses, in addition to the small NPs. The number of large NPs is increased following 150 pulses as seen in Fig. 4c. A comparison with the Au NPs on Si wafer shows that the increase in laser irradiation time leads to a more pronounced increase in Au NP size when PS is used as the substrate. Polished silicon substrates do not show this behavior as described in Section 2.1, where an increase in irradiation time does not significantly change the NP size.

**Fig. 5:**
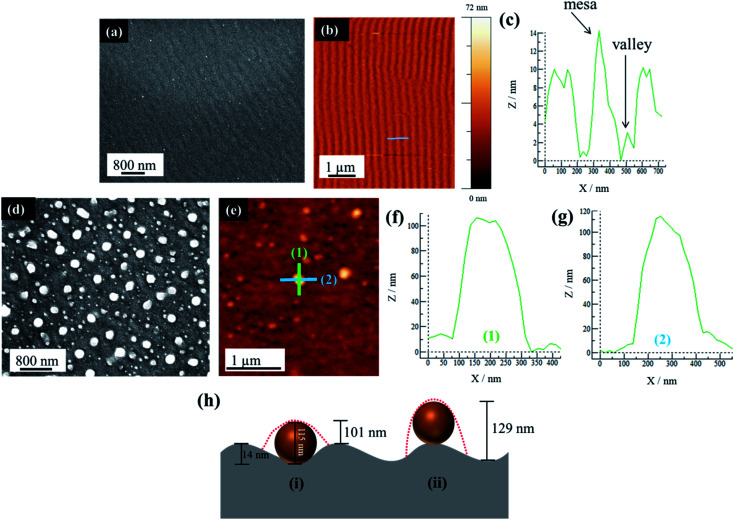
(a) FESEM of a 3.0 nm Au thin film on a PS (prepared using 3.2 wt% HF at 9.1 V for 20 min) substrate before dewetting, (b) AFM of the sample in (a), (c) corresponding line scan as indicated by the grey line in (b), (d) FESEM of a 3.5 nm Au thin film dewetted at 200 mJ cm^−2^ for 200 pulses on a PS substrate prepared using 3.2 wt% HF at 9.1 V for 20 min, (e) AFM analysis of the sample in (d) with two line scans in perpendicular directions with vertical line scan shown in (f) and horizontal line scan shown in (g), and (h) a schematic illustration indicating the difference in height profiles depending on where the NP sits, within the valley (i) or on the mesa (ii). Red dashed lines indicate profile as obtained from AFM. Not to scale.

A further examination of PS substrates shows that they possess topographical features in the form of ripples across the surface. The AFM analysis shows that the sample prior to Au deposition and PLiD illustrates ripples of a mesa-to-mesa distance of 187 ± 32 nm with a consistent mesa-to-valley depth of 14 ± 2 nm. The rippled surface is maintained following deposition of a 3.0 nm Au thin film as seen in Fig. 5a–c. Therefore, it is believed that it is the topographical features of PS substrates that result in the formation of larger NPs with increasing irradiation time. In contrast, on polished Si substrates, agglomeration due to Au mobility and Ostwald ripening are responsible for the formation of slightly larger NPs, although they never increase in diameter to the same extent as seen on PS substrates. The FESEM images of a 3.5 nm Au thin film dewetted at 200 mJ cm^−2^ for 200 pulses on a PS substrate prepared using a 3.2 wt% HF at 9.1 V for 20 min is shown in Fig. 5d along with the AFM analysis in Fig. 5e–g. Focusing on a single large NP of 115 nm in diameter, two line scans in both vertical (Fig. 5f) and horizontal (Fig. 5g) directions indicate a difference in NP height profile, at approximately 100 nm and 110 nm, respectively.

For a PS substrate prepared using 3.2 wt% HF at 15.1 V for 20 min, the rippled surface has a mesa-to-mesa distance of 187 ± 32 nm and mesa-to-valley depth of 14 ± 2 nm as mentioned previously. It should be noted that voltage does not play an important role in affecting the ripple formation. Assuming a constant mesa-to-valley depth of 14 nm, if the NP sits in the valley, a scan along the valley would reveal a height of 115 nm, *i.e.*, simply the NP diameter. However, a scan perpendicular to this would reveal a height of approximately 101 nm, *i.e.*, the NP diameter minus the mesa-to-valley depth as illustrated schematically in Fig. 5h(i). This is consistent with the AFM analysis presented in Fig. 5f–g. In contrast, if the NP were to sit on the mesa, a line scan along the mesa would reveal the NP diameter of 115 nm, but a scan perpendicular to this would reveal a height of 129 nm, *i.e.*, the NP diameter plus the mesa-to-valley depth (Fig. 5h(ii)), which is not observed using AFM. This procedure was repeated on other NPs within the sample, all resulting in the same conclusion. It should be noted that the AFM tip used in this work is 8 nm in diameter, therefore able to accurately resolve the dimensions discussed here. This AFM analysis strongly suggests that the large NPs are located within the valleys rather than on the mesas of the PS substrate.

The porous nature of the substrate induces defects in the metallic thin film and the pores act as nucleation centers that enable spontaneous voids to form upon dewetting. In addition, the chemical potential variations across the ripples of the substrate results in preferential film breakup along the mesas. This is supported by the Gibbs–Thompson relation, which expresses the dependence of local excess chemical potential, Δ*μ*, on the local curvature (*κ*), surface energy (*γ*), and atomic volume (*Ω*), as represented by eqn (1).^23,44^

1 Δ*μ* = *κγΩ*

The local curvature, *κ* = 1/*R*, with *R* being the local radius of curvature. In the case of a flat surface, *R* → ∞, therefore Δ*μ* → 0 and the topography has no effect on dewetting. For the rippled surface, there will be a positive Δ*μ* at the mesa and a negative Δ*μ* at the valley. Upon dewetting, metal atoms diffuse away from the mesa into the valleys to reduce Δ*μ*.^44,50^ In contrast, on polished Si substrates, voids form in the film due to hydrodynamic instabilities and thin film thickness fluctuations, without significant influence from the substrate topography.^31,39^

From our previous work on PS formation *via* electrochemical anodization of n-type Si, it has been shown that increasing the HF concentration leads to a decrease in pore size.^49^ The effect of HF concentration on the topography of PS surface is examined in this work. A HF concentration in the range of 3.2–12.4% wt% all lead to a rippled PS surface. However, with an increasing HF concentration, there is a decrease in both mesa-to-mesa distance and mesa-to-valley depth. As is seen in Table 1, the mesa-to-mesa distance decreases from 187 ± 32 nm to 42 ± 5 nm when the HF concentration increases from 3.2 wt% to 12.4 wt%, with the corresponding mesa-to-valley depth decreased from 14 ± 2 to 5 ± 1 nm. This relationship between HF concentration and topography are linked to the mechanism of PS formation as described in detail elsewhere.^49^

**Table 1:** Comparison of mesa–mesa distance and mesa–valley depth for PS prepared at 15.1 V, 20 min, HF concentrations from 3.2–12.4 wt% HF

HF concentration (wt%)	Mesa–mesa distance (nm)	Mesa–valley depth (nm)
12.4	42 ± 5	5 ± 1
9.4	90 ± 30	6 ± 1
6.3	123 ± 21	10 ± 2
3.2	187 ± 32	14 ± 2

**Fig. 6:**
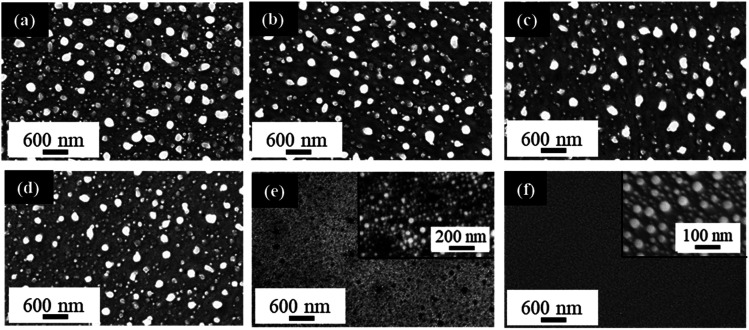
FESEM images of Au NPs fabricated by PLiD on porous silicon substrates anodized at 15.1 V for 20 min with various concentrations of HF at (a) 3.2 wt% HF, (b) 4.0 wt% HF, (c) 5.0 wt% HF, (d) 6.3 wt% HF, (e) 9.4 wt% HF with inset showing a higher magnification view of the same sample, and (f) a non-porous, polished silicon wafer substrate as a control, inset shows the same sample at a higher magnification. All samples were sputtered with 3.1 nm Au and dewetted at 200 mJ cm^−2^ for 200 pulses. All scale bars are the same.

PS fabrication and PLiD parameters were studied to reveal their effect on the formation of NPs and determine the level of tunability that is possible with the given system. The effect of HF concentration used in PS formation was first studied. Fig. 6 shows the FESEM images of Au NPs fabricated by PLiD of a 3.1 nm Au thin films on PS substrates prepared using different concentrations of HF electrolyte ranging from 3.2 to 9.4 wt%. All samples were dewetted using a laser fluence of 200 mJ cm^−2^ and 200 laser pulses. A clear difference in the morphology of produced Au NPs is observed between those on PS substrates prepared using a HF concentration lower than 9.4 wt% and that at 9.4 wt%. Specifically, formation of large NPs at the long irradiation time of 20 s (*i.e.*, 200 pulses) was only observed on PS substrates prepared with a HF concentration below 9.4 wt% (Fig. 6a–d). For PS substrates prepared with HF concentration at 9.4 wt%, this is no longer true (Fig. 6e) and a distribution of small NPs that form in relation to the pores alone is observed instead, with no influence from the topography of the substrate. Same was observed for the PS substrate prepared with a 12.4 wt% HF solution. The morphology obtained at HF concentrations above 9.4 wt% is more similar to that of a non-porous substrate as shown in Fig. 6f for comparison. Therefore, not all PS substrates result in the formation of large NPs at longer irradiation times, this behavior is only seen on PS substrates prepared with HF concentrations below 9.4 wt% HF. This is because the magnitude, size, and spacing of the rippled PS surface change with HF concentration, and as a result, influence the PLiD behavior of thin Au films on PS substrates. It also suggests that NP size can be controlled by tuning the synthesis of the PS substrate without the use of expensive and complex lithography. Furthermore, characterization of these PS substrates with no Au films present using ellipsometry and FESEM showed no significant change to the native oxide thickness and morphology, respectively, in the PS substrate following laser irradiation, indicating a minimal effect of laser irradiation on the PS substrates.

Giermann and Thompson found that substrates templated with inverted pyramids resulted in NP formation within the pits of the pyramids given appropriate thin film thicknesses and mesa widths.^23^ They noted that if the metal film is too thick relative to pit depth, NPs did not interact with the topography and formed randomly. Petersen and Mayr also showed that a suitable mesa-to-valley depth was required to enable strong interactions between the thin film and the substrate to enable diffusion of Au into the valleys.^51^ This indicates that the surface ripples must exhibit at least a 10 ± 2 nm mesa-to-valley depth in order to promote Au diffusion into the valleys, and the formation of large NPs. These conditions are met for samples prepared at 6.3 wt% HF and below (Table 1). If the ripple dimensions are less than this, they are not sufficient to facilitate the formation of large NPs in the valley (Fig. 6e). Therefore, PS samples prepared with HF concentrations below 9.4 wt% have suitable mesa-to-valley depths that enable Au diffusion into the valleys and the formation of large NPs as seen in Fig. 6a–d. Samples prepared with HF concentrations above 9.4 wt% do not meet this criteria and the formation of slightly larger NPs would be due to Ostwald ripening and Au mobility alone, with no influence from the PS substrate, mimicking the behavior of polished Si substrates instead.

It should be noted that small NPs are present on all PS samples, as they form in relation to the pores alone, which all PS samples possess. It is observed experimentally that these small NPs are located along the pore edges and within the inter-pore spaces. This behavior has also been seen in the electron-beam-induced dewetting of Au on templated, holed substrates in which the NPs formed between the holes rather than within them.^44^ EDX analysis and theoretical volume conservation using initial thin film thickness, NP diameter and NP distribution provide support that the majority of the Au thin film remains on the surface of the substrate, rather than within the pores. The volume conservation model also supports an absence of Au evaporation during laser dewetting, on either porous or polished Si substrates, as the majority of the Au deposited in thin films was found to remain. Furthermore, the pores of the substrate are too deep (66 μm at 20 min for 6.3 wt% HF and 15.1 V) to facilitate NP formation. This is advantageous for SERS applications in which surface limited Au NPs are preferred over Au deposition within the pores.^52^ In addition, material is not lost within the pores which is beneficial towards preventing the loss or inefficient use of expensive metals including Au and Pt.

**Fig. 7:**
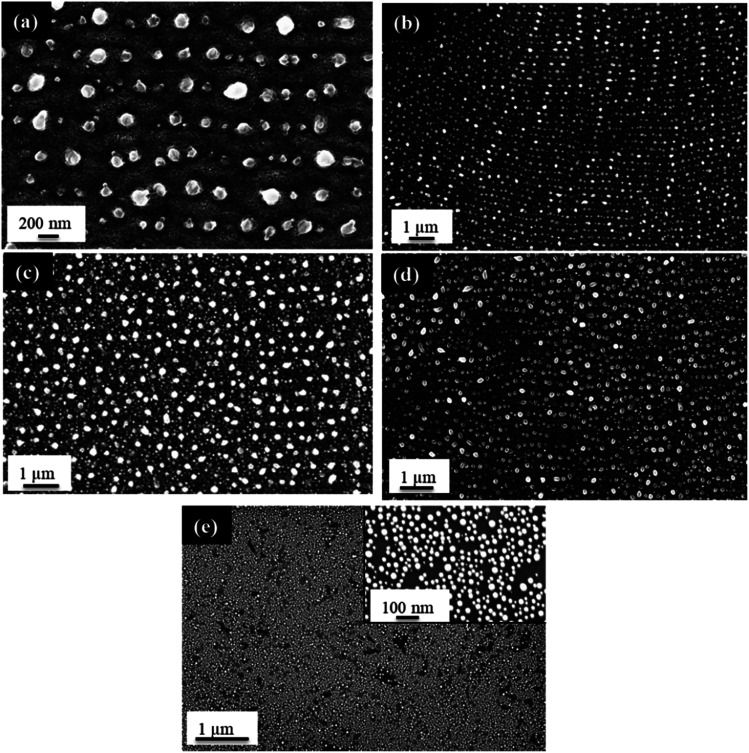
FESEM images of a 3.0 nm Au thin film dewetted at 200 mJ cm^−2^ for 200 pulses on porous silicon substrates prepared with 3.2 wt% HF for 20 min at an applied voltage of (a), (b) 9.1 V, (c) 12.1 V, (d) 16.1 V, and (e) 2.9 nm Au thin film dewetted at 200 mJ cm^−2^ for 200 pulses on polished silicon as a control with inset showing a more zoomed-in view of the same sample.

Finally, the influence of PS fabricated using different applied voltages on the Au NP formation was investigated. As described before, the applied voltage in electrochemical anodization influences the pore size of the PS, with an increase in applied voltage resulting in larger pore diameters. At a constant concentration of 3.2 wt% HF and a constant anodization time of 20 min, a range of voltages from 9.1–16.1 V was explored, with corresponding substrate mesopore diameters varying from 13 ± 3 to 21 ± 5 nm. Following PLiD of a 3.0 nm Au thin film at a constant 200 mJ cm^−2^ for 200 pulses, the diameters of the formed large NPs change from 138 ± 21 nm at 9.1 V to 185 ± 29 nm at 16.1 V, as seen in Fig. 7a–d. For comparison, a 2.9 nm Au film on a non-porous, polished Si wafer dewetted under the same conditions yields NPs of 23 ± 5 nm in diameter (Fig. 7e), being significantly smaller than when PS is utilized. When PS is prepared at 3.2 wt% HF with varying applied voltages, the magnitude of the ripples is similar. In all cases, the ripples are strong enough to influence the location and size of the large NPs, however the spatial arrangement varies with voltage. The relationship between voltage and NP arrangement is linked to pore size and inter-pore spacing. As the pores increase in diameter, the inter-pore spacing decreases. Likewise, as the pore diameter decreases, the inter-pore spacing increases. As a result, it is believed that the mesa-to-mesa spacing and mesa-to-valley depth vary slightly with voltage. This causes minor variations in NP spatial arrangement as seen in Fig. 7, although the NP diameters remain fairly constant.^23,51,53^

Larger NPs (<200 nm), as can be fabricated using the method reported here, are specifically advantageous in the catalytic synthesis of silicon nanowires for lithium ion battery (LIB) anode applications. It is known that the critical diameter for silicon nanowires is approximately 300 nm, above which they experience pulverization and degradation due to volume expansion during lithium cycling.^54–56^ In addition, nanowire growth kinetics are directly linked to catalyst diameter according to the Gibbs–Thomson effect which indicates that growth rate decreases with decreasing catalyst diameter.^57–59^ This indicates the desire for larger NPs in order to facilitate fast growth rates while remaining below the critical radius for fracture and pulverization. Due to the strong link between catalyst and nanowire diameter, the Au nanoparticles fabricated using this method would be well suited for use as a catalyst array in the subsequent silicon nanowire synthesis *via* the well-established vapor–liquid–solid mechanism for LIB anode applications. Likewise, NPs up to 200 nm in diameter have shown to be beneficial towards increasing scattering efficiency which is directly linked to an increase in NP diameter for SERS applications.^60^

On the other hand, by controlling the laser irradiation time, small NPs (9.0 ± 2.0 nm) can be attained at a single pulse, whereas larger NPs can be fabricated at longer irradiation times. Alternative non-lithographically templated substrates or masks used for PLiD such as dimpled tantalum, dimpled titanium, or porous alumina are not experimentally tunable towards small (<20 nm) NP formation, making them applicable towards a narrow or singular range of NP sizes as they are limited to the dimensions of the cavities, dimples, or mask feature size.^25,50,61^ In addition, with NP location and arrangement being tunable in relation to HF concentration and applied voltage during PS substrate fabrication, a range of catalyst arrays are achievable.^53^ The approach presented here is theoretically applicable towards all high-melting-point metals given PLiD optimization and suitable for a wide range of applications.

## 3. Conclusions

It has been shown in this work that Au NPs can be readily fabricated on both polished and porous silicon (PS) substrates using PLiD. However, the NP size and spacing depends strongly on the substrate used. On polished Si substrates, NPs of 15 ± 7 nm to 89 ± 19 nm in diameter can be fabricated by changing the initial thin film thickness. The NP size shows a linear dependence on the metal film thickness due to the mechanism of nucleation and growth of holes where the nano-island formation imposed by the Volmer–Weber growth model during the metal film sputtering process acts as the nucleation sites. It has further been demonstrated that the surface roughness of metal films can be used as an additional parameter, along with the conventionally used film thickness, to control the NP size. An increase in surface roughness leads to smaller NPs.

PS substrates introduce a topographically complex surface on which NPs form, either in relation to the pores of the substrate or in relation to the large-scale ripples. The electrochemical anodization using an electrolyte concentration in the range of 3.2 to 12.4 wt% HF all leads to a rippled surface. The ripple morphology is found to be dependent on HF concentration used. Both the mesa-to-mesa distance and the mesa-to-valley depth of the ripples show a decrease with increasing HF concentration. On PS, through control over the PLiD conditions, small NPs alone can be produced at short irradiation time, whereas an increase in irradiation time leads to a much more pronounced increase in the Au NP size produced on PS substrates due to the rippled surfaces as compared to when polished Si was used. It has been shown through AFM analysis that the larger (<200 nm) NPs are located in the valleys of the rippled PS surfaces. Furthermore, the size and spacing of ripples on the PS substrates have a significant effect on the PLiD behaviour. Specifically, the formation of large Au NPs at long irradiation time was only observed on PS substrates prepared in electrolytes with concentrations lower than 9.4 wt% HF, illustrating the need for a suitable mesa-to-valley depth to promote the formation of large NPs. At concentrations equal to or higher than 9.4 wt% HF, the mesa-to-valley depth is too small to enable the interactions between the metal thin film and the rippled substrate, leading to the Au NP formation similar to those on non-porous polished Si substrates. Overall, this fabrication method provides a simple, robust and rapid way to prepare Au NP arrays without the involvement of lithographic patterning techniques. It is theoretically applicable to a wide range of metals given the appropriate PLiD conditions are met.

## 4. Experimental methods

The PS substrate was fabricated using phosphorous-doped n-type Si (100) wafers with a resistivity of 1–20 Ω cm (Wafer World Inc.) by electrochemical etching in a solution of HF (48 wt%, Sigma Aldrich), ethanol (absolute, ≥ 99.8%, Sigma Aldrich) and deionized water (Corning Mega-Pure system). All chemicals were used as received. The thickness of the silicon native oxide layer was analyzed using a Model M2000 variable-angle spectroscopic ellipsometer (J.A. Woollam Co. Inc., USA). Electrochemical anodization was carried out in a Teflon cell where a Si sample (10 mm × 5 mm) was mounted and connected to a DC power supply (Agilent Technologies N5744A) as the anode. A cylindrical Pt mesh counter electrode (1.0 cm in diameter) was used, being positioned around the sample. A constant voltage, ranging from 9.1 to 16.1 V, was applied for 20 min. The electrolyte solutions with various HF concentrations from 3.2 to 12.4 wt% used in this work were prepared by varying the volume ratios of HF, ethanol and water. The electrolyte solution was stirred magnetically throughout the anodization and all experiments were performed without illumination at room temperature. Immediately after anodization, the samples were rinsed with copious amounts of deionized water and transferred to a vacuum desiccator.

Au thin films in the thickness range of 2.9–3.5 nm and 2.5–16.3 nm were sputter-coated on PS substrates and Si wafers, respectively, using a BAL-TEC SCD 500 sputter deposition system, with the film thickness determined by a BAL-TEC QSG 100 quartz crystal film thickness monitor. Before sputtering, the Si wafers were cleaned successively with acetone, methanol, deionized water and dried using high-purity N_2_ gas (99.999%, Praxair). The pressure of the high-purity Ar gas (99.998%, Praxair) during sputtering was controlled in the range of 4–8 mTorr. Laser radiation with a wavelength of 355 nm, the third harmonic output of a Q-switched Nd:YAG laser (Spectra-Physics, PRO-250-10) with a repetition rate of 10 Hz and a pulse width of 6–9 ns, was used to dewet the Au thin films in ambient air. The laser spot had a diameter of 7 mm, giving an illumination area of 0.38 cm^2^.

The morphologies of the PS samples after electrochemical anodization and Au NPs after PLiD were characterized using a field emission scanning electron microscope (Zeiss Sigma VP) under ultra-high vacuum conditions (<10^−10^ Torr), with an accelerating voltage of 10 kV. EDX spectroscopy was conducted using an INCA x-act EDX system by Oxford Instruments. Pore morphology was analyzed using the ImageJ software. AFM analysis was carried out using a Keysight 5500 AFM (Keysight Technologies Inc.) in contact mode with a silicon nitride cantilever (Keysight). AFM images were evaluated with WSxM image analysis software with all images filtered using a 1^st^ order fit.^62^

## Conflicts of interest

There are no conflicts to declare.

## Supplementary Material
